# Olive Fruit Refrigeration during Prolonged Storage Preserves the Quality of Virgin Olive Oil Extracted Therefrom

**DOI:** 10.3390/foods9101445

**Published:** 2020-10-12

**Authors:** Karolina Brkić Bubola, Marina Lukić, Anja Novoselić, Marin Krapac, Igor Lukić

**Affiliations:** Institute of Agriculture and Tourism, Karla Huguesa 8, 52440 Poreč, Croatia; marina@iptpo.hr (M.L.); anovoselic@iptpo.hr (A.N.); marin@iptpo.hr (M.K.); igor@iptpo.hr (I.L.)

**Keywords:** olive fruits, storage temperature, virgin olive oil, FAEE, waxes, phenolic compounds, sensory analysis

## Abstract

With the aim to investigate the influence of post-harvest olive fruit storage temperatures on virgin olive oil production parameters, composition and quality, Istarska bjelica (IB) and Rosinjola (RO) fruits were stored for seven days at room temperature (RT), +4 °C and −20 °C prior to oil production. Lower temperatures delayed post-harvest maturation of IB fruits. Theoretical oil content did not change depending on the storage temperature, while the highest oil yield and extractability index were obtained after storage at RT. Chlorophylls decreased in IB-RT and in IB-20. A decrease in the sensory quality of oils was detected after fruit storage at RT and −20 °C, while the refrigeration temperature of +4 °C preserved it. Regarding the content of fatty acid ethyl esters, an increase was observed in IB-RT oils. Storage at RT increased the content of waxes, while the lower temperatures partially suppressed this phenomenon. In oils of both cultivars, storage at +4 °C preserved the concentration of most phenolic compounds at a level more similar to that of the fresh oil when compared to the other two treatments. In the production conditions, when prolonged fruit storage is necessary, refrigeration seems to be the most suitable option.

## 1. Introduction

Olive oil production is a seasonal activity, and as such faces particular problems, which are more or less pronounced depending on the year-by-year situation, such as the global and micro climate conditions (rainfall and temperature), early or late ripening, decreased or surplus production (olive and oil yield) and even market positioning. Such factors affect harvesting decisions and may lead to inadequate/inefficient/insufficient oil production capacity in olive mills. Virgin olive oil (VOO) is obtained by several mechanical and physical processes, which begin with harvesting and post-harvest storage of olive fruits. The quality of VOO is closely related to the quality of olive fruits from which it is obtained [1]. In order to achieve high quality oil, it is recommended to process olives within 24 h after harvesting [2]. If processing is not done within that period, as for example when the capacity of available mills is insufficient, the fruits have to be stored for a certain period of time.

During a prolonged storage period, degenerative hydrolytic and oxidative processes in olive fruits start to develop. Under relatively high storage temperatures the proliferation of various microorganisms in olive fruit is accelerated, which often leads to many detrimental changes in olive physicochemical composition and the development of sensorial defects of fermentative origin, in the obtained olive oil [3,4].

Besides volatile compounds, principally associated with a decrease in olive oil sensory quality caused by prolonged olive fruit storage [4], other quality markers are affected as well. In olives with ruptured drupe (unhealthy, overripe, fractured during picking or storage), the fatty acids are liberated by the action of enzymes. In the presence of ethanol, also a product of fermentation, fatty acid ethyl esters (FAEE) are formed by esterification of free fatty acids with ethanol [5,6]. Recent studies suggest that the FAEE concentration is considered an indicator of olive oil quality [7,8]. Higher storage temperatures, such as the ambient temperature of 25 °C, accelerate the processes of olive ripening [9]. As olive fruit ripens, its exocarp becomes thinner, and fruit tissues become softer [10]. Along with these changes, particular compounds are being extracted into oil, such as waxes from the waxy surface layer of the cuticle of olive fruit. The content of waxes in the oil can also be considered a quality indicator [6] although it is officially used as a marker for distinguishing olive from olive-pomace oil [8,11]. Phenolic compounds, in addition to their involvement in VOO taste characteristics, have antioxidant properties and, therefore, significantly contribute to the oxidative stability and health benefits associated with VOO [12]. Their presence in oil depends on the interaction between genetic (cultivar), environmental (cultivation, harvest and postharvest conditions) and physiological factors (fruit ripening degree and sanitary conditions), and processing conditions [13,14]. Previous studies revealed that phenolic compounds are strongly affected by olive fruit storage conditions, especially secoiridoids, whose concentrations decrease with longer storage times and higher storage temperatures [15,16]. Several authors have found that, due to genetically predetermined enzymatic activity, the polyphenol content of obtained VOO behaved differently among cultivars even if the fruits were stored under the same conditions prior to oil production [16,17]. Considering all the reasons mentioned above, optimal duration and temperature of fruit storage are obviously very important factors for obtaining high quality VOO in general [9] and are also crucial for preserving particular sensory profiles typical for VOO of certain cultivars, consisting of high levels of phenolic compounds and related high intensities of bitterness and pungency, as in the case of Croatian autochthonous Istarska bjelica and Rosinjola [18,19].

Currently, the main strategies for avoiding deterioration of olive fruit during storage and production of lower quality VOO include reducing the period between harvesting and processing by increasing the production capacity of olive mills, as well as applying conditions aimed to ensure lesser contact between fruits (larger storage spaces, perforated boxes, etc.) [20]. Storage of olive fruits at lower temperatures was also recognized as an alternative that could allow more flexibility during harvesting and oil production [17]. In general, low storage temperatures decrease water activity and inhibit microbial growth [21], slow down enzymatic and biochemical reactions [22], decrease fruit respiration and delay harvested fruit maturation [10,23]. On the other hand, low temperatures may cause physiological damage of the fruit [24] since ice crystals formed during frozen storage may break down the cell structure and at the same time allow the contact between various enzymes and substrates that could affect VOO quality [25,26]. Very low temperatures during olive fruits development and harvest, with related cycles of freezing and defrosting of fruits on the tree, are known to cause biochemical changes, which significantly modify its volatile compound and phenolic profiles [22] and could induce the development of the “frostbitten olives” sensory defect in olive oil [27].

Several studies have been conducted in order to investigate appropriate olive fruit storage conditions, including mostly ambient and refrigeration temperatures [9,16,21,23,28]. A few studies that examined the effects of frozen storage included either a very short storage period of one day [29], which might not be sufficient in practical conditions when delays in olive processing are larger, or a very long storage period of 6 months [26], which is not applicable from a practical point of view. These studies focused mostly on the basic quality parameters and phenolic and volatile profiles of the obtained oils and, to our knowledge, none of them investigated the influence of low storage temperatures on the contents of FAEE and waxes.

The aim of this study was to investigate the influence of low temperature during prolonged post-harvest storage of olive fruit on the production parameters (oil content in the fruits, oil yield and extractability index), composition (the content of pigments, FAEE, waxes and phenolic compounds) and sensory quality of olive oil obtained therefrom. The experiment was performed with olive fruits of two autochthonous Croatian cultivars, Istarska bjelica and Rosinjola, non-stored (control) and stored for seven days at three different temperatures, including room temperature, refrigerating at +4 °C and freezing at −20 °C. The main intention was to evaluate the possibility of prolonging the storage period of olive fruits to a reasonable time still feasible in practice (one week), without compromising the chemical and sensory quality of VOO, which would possibly contribute to overcoming the problems with olive mill overloading during the harvesting period. In addition, particular attention was devoted to the influence of olive fruit storage temperature on both positive and negative sensory characteristics of the obtained VOO, in order to deepen the understanding of the origin of some VOO defects, such as “frostbitten olives”, otherwise associated with the impact of low ambient temperatures during olive fruit maturation.

## 2. Materials and Methods

### 2.1. Olive Fruits, Storage Treatments and Virgin Olive Oil Production

Healthy olive fruits from Istarska bjelica (IB) and Rosinjola (RO), two Croatian autochthonous olive cultivars (*Olea europaea* L.), were manually harvested at the end of October 2016 and the beginning of November 2016, respectively. Both cultivars were grown in the same experimental olive orchard of the Institute of Agriculture and Tourism (Poreč, Croatia). The ripening index of olive fruits was determined by the protocol described by Beltran et al. [30].

Olive fruits were divided in twelve batches of 3 kg per cultivar. Three batches per cultivar were processed into oil immediately after harvest (control oil), and the rest of the olive fruits was stored for seven days at three different temperatures prior to olive oil production: at room temperature of 22 ± 4 °C (RT), at +4 °C in a refrigerator and at −20 °C in a freezer. The olive fruits stored at 4 °C and −20 °C were allowed to reach room temperature before milling, which lasted about 2 and 6 h, respectively. Fruits were crushed by a hammer crusher and olive paste was malaxed for 45 min at 25 °C using vertical thermostated olive paste mixers. Olive oil extraction was done by a laboratory centrifuge (Abencor, MC2 Ingeneria y Sistemas, Seville, Spain). In order to obtain enough oil for analysis, fruits representing one batch of 3 kg, were processed in triplicates and obtained oils were mixed in one oil sample. Obtained oil samples (*n* = 12 per cultivar, 3 control oils and 3 samples per each storage time/temperature) were left to sediment naturally for ten days and were then decanted. The analyses of oil samples started immediately after decantation. Samples were stored in non-transparent bottles at 16–18 °C during the time of analysis.

### 2.2. Oil Content, Oil Yield and Extractability Index

Theoretical oil content in the fruit (expressed on fresh and on dry weight based on the gravimetric determination of water in fruit) was determined from the olive paste obtained after crushing using Soxtec Avanti 2055 apparatus (Foss Tecator, Höganäs, Sweden) according to the method described by Brkić et al. [31].

Oil yield (%) was calculated from three parallel processing repetitions, multiplying by 100 the mass ratio of mechanically extracted oil (g) and centrifuged olive paste (g) [32].

Olive oil extractability index (EI) was calculated according to Beltran et al. [30] using the formula: EI = V × d/W × F × 100, where V (mL) is a volume of olive oil extracted, d (0.915 g/mL) is the average olive oil density, W (g) is olive paste weight and F (%) is the oil content of the fruit (on fresh weight).

### 2.3. Analysis of VOOs Pigments

Chlorophyll and carotenoid concentrations were determined using a Varian Cary 50 UV/Vis spectrophotometer (Varian Inc., Harbour City, CA, USA) following the procedure of Mínguez-Mosquera et al. [33] and expressed as pheophytin a and lutein content, respectively.

### 2.4. Sensory Analysis

Quantitative descriptive sensory analysis of VOO samples was performed by the Panel for sensory assessment of VOO, accredited for VOO sensory analysis according to the EN ISO/IEC 17025:2007 and recognized in continuation by the International Olive Council (IOC) from 2014. The panel consisted of eight assessors (5 female, 3 male, average age 35) trained for VOO sensory analysis according to the IOC method [34].

### 2.5. FAEE and Waxes

FAEE and waxes were determined by the IOC method [11] employing extraction by column chromatography and analysis by gas chromatography (GC) with flame-ionization detection using a Varian 3350 gas chromatograph (Varian Inc., Harbour City, CA, USA).

### 2.6. Analysis of Phenolic Compounds

Extraction and HPLC analysis of phenolic compounds using an Agilent Infinity 1260 System (Agilent Technologies, Santa Clara, CA, USA) in oil samples was performed according to the method proposed by Jerman Klen et al. [35] and slightly modified by Lukić et al. [36].

Identification of peaks was performed by comparing retention times and UV/Vis spectra with those of pure standards and those from the literature [35]. The detection was carried out at 280 nm for simple phenols, lignans, secoiridoids and vanillic acid, at 320 nm for vanillin and *p*-coumaric acid, and at 365 nm for flavonoids. For quantification, standard calibration curves were made for tyrosol, hydroxytyrosol, vanillic acid, vanillin, *p*-coumaric acid, luteolin, apigenin, pinoresinol and oleuropein. Based on constructed calibration curves, concentrations of samples were expressed as mg/kg oil. Semiquantitative analysis was performed for hydroxytyrosol acetate, acetoxypinoresinol and secoiridoids, where the concentration was expressed as hydroxytyrosol, pinoresinol and oleuropein, respectively, assuming a response factor equal to one. Total phenolic content was presented as the sum of all the identified phenolic compounds.

### 2.7. Data Elaboration

To investigate the effects of different fruit storage temperature on the VOO’s investigated parameters, results of the chemical and sensorial analysis were subjected to a one-way analysis of variance (ANOVA). Means were compared by the Tukey’s honest significant difference test at the level of *p* < 0.05. Statistical analysis was carried out using Statistica v. 13.2 software (Stat-Soft Inc., Tulsa, OK, USA).

## 3. Results and Discussion

### 3.1. Oil Content, Extractability Index and Ripening Index

In order to monitor the accumulation of oil in olive fruits during storage, oil content on dry matter was determined (Table 1). It was determined that it did not change significantly depending on the fruit storage temperature in the case of both investigated cultivars. This result indicates that the accumulation of oil did not continue during fruit storage, which is in agreement with the findings of Inarejos-García et al. [37] during the storage of Cornicabra cultivar fruits at 10 °C and 20 °C for three weeks, and that of Yousfi et al. [23] in the case of Arbequina olives stored up to three weeks at 3 °C and 18 °C.

On the other hand, considering the processing parameters, olive oil yield and extractability index (EI), the highest values were obtained in the case of storage at RT (Table 1). Yousfi et al. [10] reported that olives stored under ambient temperature (18 °C) exhibited higher respiration rates than refrigerated ones, which is associated with fruit ripening and, consequently, softening. As a consequence of ripening, degradation of walls of oil-bearing cells is facilitated and the extraction process is improved, which could have been the cause of the increase of the olive oil yield and EI in the RT stored fruits in this study. In IB fruits, a significant increase in RI was observed after seven days of storage and it depended on the temperature, since it increased only in the case of fruits stored at RT. In RO fruits significant differences between the treatments were not found (Table 1). García et al. [38] have also found that cold storage (5 °C) could delay ripening of Blanqueta and Villalonga olives compared to storage at ambient temperature (12 ± 5 °C). Different from the results of this study, the extractability of Arbequina olives stored up to 21 days at 3 °C and 18 °C showed a similar oil yield to the initial unstored sample [23]. Extractability index is highly dependent on the cultivar and its fruits properties [30]. Both of the investigated autochthonous Croatian cultivars had the value of EI in line with most of the leading Spanish olive cultivars [30], indicating their good potential for oil production regardless of the storage temperature of the fruit prior to processing.

### 3.2. VOO Pigments

**Figure 1:**
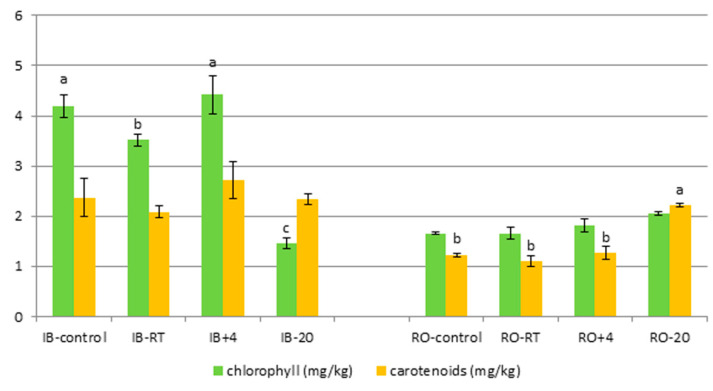
Concentrations of chlorophyll and carotenoids (mg/kg) in Istarska bjelica (IB) and Rosinjola (RO) monovarietal virgin olive oils obtained from fresh fruits immediately after harvest (control) and oils obtained from fruits stored seven days at three different storage temperature (RT—room temperature, +4 °C and −20 °C) prior to production. Results are expressed as mean values ± standard deviation of three technical repetitions. Mean values labeled with a different letter, within one parameter and one cultivar are statistically different (Tukey’s test, *p* ˂ 0.05). In case there were no statistically significant differences the letters were omitted.

Considering the chlorophyll content (Figure 1) in the VOO obtained from IB fruits, similar content was determined in IB+4 as in IB-control oil, while a mild decrease in IB-RT and a pronounced decrease in IB-20 compared to IB-control oil was determined. García et al. [39] have found that the maturation of Picual cultivar olive fruits was delayed while stored at 5 °C or 8 °C, compared to oils obtained from fruits stored at ambient temperature. The cause of this was low temperature, which delayed the destruction of chlorophyll pigments and their substitution by anthocyanins in the cells of olive skin during fruit maturation [39]. On the other hand, Morelló et al. [40] have found a decrease in the content of pigments (chlorophylls and carotenoids) in Arbequina oils obtained from fruits that were frozen on the trees seemingly due to the activity of chlorophyllase enzymes involved in the loss process. By visual inspection it was observed that the chilling injuries of IB-20 fruits occurred in the form of browning, which probably influenced a decrease of chlorophylls in the obtained oils. Chlorophyll content was also low in the oil obtained from Koroneiki olives after 30 days of storage at 0 °C, probably due to chilling [41]. The oil from olives stored at 5 °C had slightly lower chlorophyll content, while the oil from olives stored at 7.5 °C had similar chlorophyll content as the oil from freshly harvested olives [41]. A significant effect of different storage temperature on the chlorophyll content was not observed in RO oils, probably because its fruits did not continue to ripen during storage (Table 1), while fruit injuries as a result of freezing during storage were not observed by visual inspection. Yousfi et al. [10] also found that storage conditions (3 °C and 18 °C during 3 weeks) did not affect the content of chlorophylls in Arbequina fruits.

Carotenoids content was not significantly changed depending on the fruit storage temperature in both IB and RO oils, except in the case of RO-20 oil where an increase was detected when compared to RO-control oil (Figure 1). Yousfi et al. [10] have found that carotenoids were not affected by the storage conditions (3 °C and 18 °C during 3 weeks) applied to the Arbequina fruits. The increased content of carotenoids in RO-20 could be related to a decrease in the consistency of the chloroplast wall caused by low storage temperature that facilitates the release of these pigments into olive oil [23].

### 3.3. Sensory Quality

**Figure 2:**
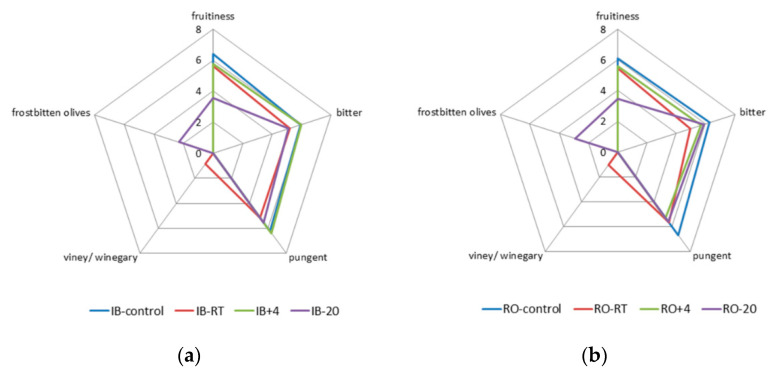
Results of sensory analysis of (**a**) Istarska bjelica (IB) and (**b**) Rosinjola (RO) monovarietal virgin olive oils obtained from fresh fruits immediately after harvest (control) and oils obtained from fruits stored seven days at three different storage temperature (RT—room temperature, +4 °C and −20 °C) prior to production. Results are expressed as mean values of the medians of three technical repetitions.

After fruit storage, panelists observed a decrease in fruitiness and a major positive aroma sensory characteristic in the oil samples of both cultivars, (Figure 2). The highest decrease was determined in oils stored at −20 °C (approximately 3 intensity units compared to control oils). The taste characteristics of the oils, such as bitterness and pungency, were less altered after the prolonged fruit storage than the olfactory characteristics, although in most cases slightly lower intensities were determined compared to the control, except for IB+4 oil, which was similar to IB-control oil (Figure 2). García et al. [39] found that bitterness and sensory quality of Picual oils obtained from fruits stored at RT decreased rapidly and that the loss was slowed down during storage at 5 °C. Morelló et al. [40] found that Arbequina olive oil had a decreased intensity of bitterness and pungency when produced from fruits that have been frozen on the trees. Inarejos-García et al. [37] observed a larger reduction of bitterness, determined as K225, in Cornicabra olive oil produced from fruits stored for 5 days at 20 °C compared to that obtained from olives stored at 10 °C for a week. The same authors concluded that prolonged storage could be useful for modifying the taste of oils of phenol-rich cultivars, such as Spanish Cornicabra, characterized by intense bitter taste that could affect consumers’ preferences. On the other hand, preserving the bitterness and pungency in IB and RO oils could be very important, since the mentioned sensorial characteristics were shown to be typical for these autochthonous monovarietal olive oils [18,19], especially because these cultivars are included in the production of Croatian oils under the protected denomination of origin (PDO) “Istra”, which gives them an added value.

In the oil samples obtained from the fruits stored for seven days at RT and −20 °C negative sensory characteristics were determined (Figure 2). In RT oils of both cultivars a slight intensity (around 1) of the “viney/winegary” defect was noted, while the defect “frostbitten olives” was recognized as the main defect in the oils obtained from fruits stored at −20 °C, with the intensity of 2.3 in the case of IB and 2.9 in the case of RO cultivar oil respectively. IB and RO oils obtained from the fruits stored at RT and −20 °C could not be classified as extra virgin olive oils (the highest quality category), since, according to the intensity of the recognized defects, they belonged to the virgin olive oil category (EEC, 1991). Sensory defects were prevented by the storage at +4 °C (Figure 2), indicating that +4 °C was the most appropriate temperature for fruit storage in order to assure good sensorial quality of the obtained oils. “Viney/winegary” defect, and “fusty”, “muddy sediment” and “musty” defects usually develop in oils because of the proliferation of particular microorganisms (lactic, acetic and enteric bacteria, fungi and *Pseudomonas*) on olive fruits during unsuitable storage conditions [3,4]. Kiritsakis et al. [41] reported that Koroneiki olives stored at 0 °C and 5 °C had no fungus development, while this was not the case at 7.5 °C, where the noticed increase in oil acidity was a result of fungal lipase activity [41], which can cause development of sensory defects. Garcia et al. [38] have found a different response of the sensory quality of different cultivars: Blanqueta olive oil developed defects more rapidly than Villalonga olive oil during 30 days of storage at ambient temperature and at 5 °C, and the development of off-flavors was more rapid at ambient than at low temperature.

Freeze injuries are a consequence of olive fruit cell dehydration and destruction caused by ice crystals forming inside the parenchyma cells, which cause destruction of cell membranes, leading to cell death and high oxidation of cell contents [40]. This is the consequence of the contact between enzymes and their respective substrates, which may have an effect on the composition of the obtained olive oil [40]. Freeze injuries were not detected on olives of both cultivars stored at +4 °C, which is in agreement with the result for Koroneiki olives stored at 5 °C and 7 °C for 40 days [41]. On the other hand, freeze injuries in the form of fruit skin browning and shriveling were detected by visual inspection on olives stored at −20 °C, which finally resulted in olive oils with perceived “frostbitten olives” defect (Figure 2). Although some authors reported that severe freezing damage of olive fruits on trees during winter time could have negative influence on the sensory characteristics of olive oil [42,43], there is little information on how controlled freezing temperatures during olive fruit storage influence the sensorial profile of obtained oils. Romero et al. [43] reported two different descriptions of the “frostbitten olives” defect, which depend on whether the temperature changes took place abruptly, with rapid freeze–thaw cycles, or gradually. They reported that oils were grouped based on the concentrations of volatile compounds into two clusters, characterized by different profiles. The first was characterized by descriptors such as “soapy” and ”strawberry-like” and the characteristic presence of ethyl 2-methylbutanoate and ethyl propanoate, and the second by “wood” and “humidity” descriptors and high concentrations of pentanal and octanal. In this study, the sensory profile of the “frostbitten olives” defect perceived by the panelists was described using a descriptor “wet wood” (Figure 2), which was more similar to the second profile reported by Romero et al. [43], indicating that a gradual drop of temperature took place during the controlled freezing at −20 °C, with the formation of extracellular ice and evaporation of liquid water inside the cells. According to Romero et al. [43], as water is removed from the cells, ice continues to grow and damages the cells until they break down.

### 3.4. FAEE and Waxes

FAEEs are closely related to health conditions of the fruits and their concentration is higher in olives that underwent hydrolytic and fermentative processes that produce additional amounts of free fatty acids and alcohols [44]. Regarding the FAEE parameter, there were no significant differences among the treatments in RO oils. However, an increase in FAEE concentration was observed in IB-RT compared to the IB-control oil (Table 2), which was probably a result of the softening and damage of the fruit tissue during prolonged storage as a consequence of accelerated ripening of the fruits at higher storage temperature (Table 1). Jabeur et al. [44] have found an increase in FAEE concentration during Chemlali olive fruit storage at ambient temperature (12–18 °C) for 25 days in closed plastic bags and in open perforated plastic boxes, probably a consequence of microorganism fermentation activity. In the oil samples investigated in this study, total FAEE concentration ranged from 4 to 12 ppm and as such was below the maximum legal limit of ≤35 ppm set for EVOO [8]. Although the FAEE values did not surpass the maximum legal limit, they were in line with the results obtained by sensory analysis of the IB-RT oil, where a slight intensity of “viney/winegary” defect was determined (Figure 2). The correlation found between FAEE amounts and fermentative defects was probably due to their common origin [1,6]. On the other side, the intensities of non-fermentative defects, e.g., “frostbitten olives”, determined in the oils obtained after frozen storage of the fruits of both cultivars (Figure 2), are not related to the concentrations of FAEE as reported by the literature [1].

The concentration of waxes (C246) in the investigated samples ranged from 15 to 50 ppm (Table 2). Although the obtained values did not surpass the maximum legal limit for EVOO of ≤150 ppm [8], the RT treatment showed a significant increase in the concentration of most waxes compared to the controls and the other two treatments in the oils from both cultivars. Storage at room temperatures may cause acceleration of fruit ripening [9], which is followed by fruit cuticle thinning and softening of fruit tissue [10]. As a consequence of those changes, waxes from the waxy surface layer of the cuticle of olive fruit could be more easily extracted into oil. The more mature, and possibly the more degraded olive fruits were (as in the case of IB fruits stored at RT, Table 1), the higher was the amount of waxes extracted, which supported the assertion that higher concentration of waxes could indicate lower quality of olive oil [6,44]. The storage of fruits at temperatures lower than RT resulted in lower concentration of waxes in the obtained oils (Table 2), probably due to the delay in fruit ripening.

### 3.5. Phenolic Compounds

A reduction of the total concentration of the identified phenolic compounds was detected in the oils from particular treatments, but the highest decrease compared to the control treatment was detected in the oils obtained from the fruits of both cultivars stored at −20 °C (Table 3). Hachicha Hbaieb et al. [9] have found that negative effects of storage time on phenolic compounds in oils were enhanced by an increase in storage temperature from 4 to 25 °C. Yousfi et al. [10] found that the main phenolic compounds in VOO exhibited a reduction during 15 days of fruit storage, which was in correlation with the increase in the applied temperature (from 2 to 18 °C). Other authors, who investigated the influence of freezing of fruits on trees, reported a decrease in the concentration of phenolic compounds in the obtained oils. They explained it as a consequence of fruit freeze injuries, which lead to cell dehydration and destruction of cell membranes, and consequently to cell death and high oxidation of cell contents as a result of the contact between enzymes and their respective substrates, which might have affected the phenolic composition of the oils [40,42]. Morelló et al. [40] investigated the influence of freezing of Arbequina fruits on trees on phenols in the obtained oils. They have found that total phenols and secoiridoids decreased after frost because ice crystals destructed olive tissues, which encouraged the oxidative degradation of phenolic compounds in reactions catalyzed by polyphenol oxidase enzyme [40]. Masella et al. [26] investigated the difference between three different methods of freezing of olives and found a significant reduction of total phenols in oils obtained from fruits after 6 months of storage at freezing temperatures (about 40% of the control oils) regardless of the freezing method used. It must be mentioned that not all the identified phenolic compounds absorb UV light equally, meaning the use of oleuropein as a standard for all secoiridoids with the response factor equal to one in the HPLC-DAD analysis in this study might have resulted with an overestimation of the reduction of the total phenol concentration in the oils of particular treatments. For example, p-HPEA-EDA (oleocanthal) has a lower response factor in comparison to 3,4-DHPEA-EDA (oleacein), and the same applies for the corresponding aglycone isomers of ligstroside and oleuropein. This difference is related to the different substitution of the aromatic ring. The underestimation of the secoiridoids bearing the tyrosol moiety might have had a notable impact on the calculated total phenol concentrations. More specifically, although the reduction noted is relative for each compound, the actual total phenolic loss might be less than reported.

Considering the secoiridoid group, a reduction was found in the case of treatments at RT and −20 °C in the oils from both cultivars. Since secoiridoid compounds are strongly related to the VOO shelf life [45], it can be assumed that the oils stored at +4 °C would have the longest shelf life among the oils obtained from the stored fruits. Reduction of secoiridoids was lower in RO oils, which initially had a lower concentration of total secoiridoids compared to IB oils. Li et al. [15] noticed that the higher the initial concentration of these phenolic compounds in oil, the faster they decrease during storage, possibly because higher concentrations are more susceptible to oxidation with respect to other antioxidants in olive oil. Guillaume et al. [42] noted a reduction of the concentration of secoiridoids in the oils obtained from the frost-damaged fruits of three olive cultivars (Frantoio, Barnea and Picual) grown in Australia. Hachicha Hbaieb et al. [9] have also observed a larger decrease in secoiridoids concentration in Arbequina and Chétoui oils obtained from fruits stored at 25 °C than at 4 °C, and related this to the lower β-glucosidase activity determined in olive fruits from the former treatment.

Considering the particular secoiridoid compounds in IB oils, most of the concentrations decreased in the oils obtained from stored fruits. Quantitatively the highest reduction with respect to IB-control was determined for the concentration of 3,4-DHPEA-EDA in IB-RT and IB-20 oils. In RO oils from the stored fruits the highest decrease with respect to RO-control oil was detected in the case of 3,4-DHPEA-EDA and oleuropein aglycone (isomer I) after fruit storage at −20 °C and at RT In the oils from both cultivars obtained from fruits stored at +4 °C the profile of secoiridoids was more similar to the control oils than that of the other two treatments. It is probable that the cold storage conditions slowed down the rate of enzymatic and biochemical reactions, which lead to the degradation of these particular phenols, as noticed in RT oils, and at the same time avoided the negative effects caused by freezing, observed in the oils obtained from the fruits stored at −20 °C. These findings are in agreement with the results of Hachicha Hbaieb et al. [9], who found more similar phenolic profiles of the oils obtained from Arbequina fruits stored at 4 °C and the freshly harvested ones, in comparison to the oil obtained from fruits stored at 20 °C, which was explained by the similar endogenous enzyme activity patterns detected in the fruits of the former treatments. Romero et al. [27] characterized the phenolic profile of Spanish olive oils (Cornicabra, Hojiblanca, and Picual cultivars) with “frostbitten olives” sensory defect and found that the concentrations of all the investigated groups of phenols decreased in defective oils, except secoiridoids. The authors [27] explained these differences by considering the action of enzymes that are affected by frost; physical damage of olive fruits by ice crystals formed during freezing leads to cellular destruction, allowing phenolic substrates to mix with polyphenol oxidase (PPO), which degraded them. In this study, lower concentrations of the majority of phenols, even secoiridoids, were found in oils obtained from the fruits frozen at −20 °C in comparison to control oils. This was probably due to the controlled freezing process applied, which did not include freezing and thawing cycles that would correspond to those occurring naturally in the olive orchard.

In both cultivars, a significant increase in the concentrations of simple phenolic compounds, hydroxytyrosol and tyrosol, was found in the oils obtained from the fruits stored at RT and +4 °C compared to the control oils (Table 3). The increase observed was proportional to the storage temperature applied, which was as expected, since it can be explained by increased hydrolysis of complex phenols into simple phenols at higher temperatures [15]. On the other hand, after storage at −20 °C, no significant change in hydroxytyrosol and tyrosol concentrations was found when compared to the control oils. Such an outcome could have possibly been connected to partial inactivation or the lower ability of PPO and peroxidases (PODs) to oxidize biophenolic glucosides at lower storage temperature, as reported earlier [46].

The concentration of total lignans decreased in the case of both monovarietal oils obtained from fruits at −20 °C, which was in agreement with the findings of Masella et al. [26], while in IB+4 oil the increase of total lignans was mainly a consequence of an increase in pinoresinol concentration. Guillaume et al. [42] reported that the concentration of lignans was strongly positively correlated with the intensity of the “frostbitten olives” defect, and that the concentration of acetoxypinoresinol increased after the freezing of olive fruits on the trees, which was not confirmed by the findings of this study in the case of controlled frozen storage.

The concentration of total flavonoids decreased in all the IB treatments, while for RO a decrease was detected only in the case of RO-20 oil. In the oils of both cultivars obtained after storage of fruits at +4 °C the profile of individual flavonoids was more similar to the one observed in the control oils than in the other two treatments. Other authors reported different trends in flavonoids behavior under various storage conditions. Hachicha Hbaieb et al. [9] reported higher flavonoids content in the oils extracted from olives stored at 4 °C than at 20 °C, probably due to the accelerated process of ripening of fruits at the higher temperature. The content of flavonoids in Cornicabra oils obtained from fruits stored at 10 °C and 20 °C did not show a clear trend at the beginning of storage, probably because of their stable structure and high oxidation resistance, while an increase of particular flavonoids was determined after a prolonged storage, probably because of the destruction of the cell structure and the release of bound phenols [37].

The concentration of total phenolic acids only increased after the RT treatment in oils of both cultivars, as a consequence of a sharp increase in p-coumaric acid concentration. Storage at lower temperatures had no influence on the concentration of phenolic acids, which was not in agreement with the results of Masella et al. [26], who reported a decrease in the concentration of p-coumaric acid after 6 months of frozen storage of olive fruits. The discrepancy observed was possibly related to the difference in storage time between the two studies.

## 4. Conclusions

The results of this study have shown that, when conducted at an appropriate temperature, storage time of olive fruits can be prolonged to seven days without compromising the crucial aspects of olive oil quality. Although prolonged storage at room temperature increased the oil extractability index, this treatment exhibited many serious drawbacks, such as the elevated concentrations of fatty acid ethyl esters and waxes, loss of a certain proportion of valuable phenolic compounds and the occurrence of sensory defects in the obtained oil, most probably due to fermentative processes induced by accelerated post-harvest fruit ripening. Prolonged storage at the freezing temperature of −20 °C also resulted in significant alterations in the composition and quality of the obtained oil, including a decrease in the concentration of phenols and generation of the “frostbitten olives” sensory defect, presumably induced by freezing injuries and modified enzymatic activity in the fruits. The treatment that included prolonged refrigeration of fruits at +4 °C proved to be the most suitable for this purpose, since it preserved the composition and sensory quality most similar to that of the fresh oil of the control treatment, which corresponded to the highest quality category, extra virgin olive oil. The results obtained point to the need to improve olive fruit post-harvest storage technical capabilities and conditions, in order to prevent losses in olive and olive oil quality and value in situations when the harvested amount of fruit exceeds the processing capacity of available mills.

## Figures and Tables

**Table 1:** Ripening index (RI), oil on dry weight, yield and extractability index (EI) of Istarska bjelica (IB) and Rosinjola (RO) cultivar fresh fruits immediately after harvest (control) and fruits stored seven days at three different storage temperature (RT—room temperature, +4 °C and −20 °C) prior to production.

	RI	% Oil on Dry Weight	Yield (%)	EI
IB-control	1.02 ± 0.10 ^b^	40.30 ± 1.60	10.03 ± 0.20 ^b^	0.45 ± 0.02 ^b^
IB-RT	1.73 ± 0.07 ^a^	37.95 ± 3.77	11.37 ± 0.38 ^a^	0.55 ± 0.04 ^a^
IB+4	1.11 ± 0.05 ^b^	40.84 ± 1.02	9.88 ± 0.17 ^b^	0.44 ± 0.00 ^b^
IB-20	1.16 ± 0.10 ^b^	40.90 ± 2.57	9.19 ± 0.42 ^b^	0.41 ± 0.03 ^b^
RO-control	1.64 ± 0.07	35.53 ± 2.76	5.11 ± 0.21 ^b^	0.22 ± 0.01 ^b^
RO-RT	1.75 ± 0.05	38.63 ± 4.51	7.14 ± 0.15 ^a^	0.29 ± 0.03 ^a^
RO+4	1.72 ± 0.06	35.75 ± 1.95	5.25 ± 0.21 ^b^	0.23 ± 0.02 ^b^
RO-20	1.58 ± 0.11	39.37 ± 3.00	5.33 ± 0.16 ^b^	0.21 ± 0.02 ^b^

Results are expressed as mean values ± standard deviation of three technical repetitions. Mean values labeled with a different superscript letter, within the same column and same cultivar are statistically different (Tukey’s test, *p* ˂ 0.05). In case there were no statistically significant differences the letters were omitted.

**Table 2:** Concentrations of ethyl esters and waxes (mg/kg) in Istarska bjelica (IB) and Rosinjola (RO) monovarietal virgin olive oils obtained from fresh fruits immediately after harvest (control) and oils obtained from fruits stored seven days at three different storage temperature (RT—room temperature, +4 °C and −20 °C) prior to production.

	Ethyl Esters			Waxes					
	EE C16	EE C18	FAEE	C40	C42	C44	C46	C246	C0246
IB-control	2.58 ± 1.53	1.95 ± 0.87 ^c^	4.53 ± 1.8 ^b^	12.59 ± 0.76 ^c^	9.54 ± 0.98 ^c^	2.59 ± 0.22 ^b^	2.92 ± 0.72	15.06 ± 1.28 ^c^	27.64 ± 1.82 ^c^
IB-RT	3.48 ± 1.04	8.24 ± 0.82 ^a^	11.71 ± 0.28 ^a^	21.13 ± 1.66 ^a^	15.08 ± 0.95 ^a^	3.42 ± 0.15 ^a^	3.29 ± 0.25	21.79 ± 0.71 ^a^	42.92 ± 2.37 ^a^
IB+4	2.71 ± 0.53	4.38 ± 1.14 ^b^	7.09 ± 1.61 ^b^	17.28 ± 0.42 ^b^	10.48 ± 0.27 ^bc^	2.67 ± 0.11 ^b^	2.37 ± 0.25	15.52 ± 0.39 ^c^	32.81 ± 0.80 ^b^
IB-20	2.64 ± 0.12	3.56 ± 0.68 ^bc^	6.21 ± 0.56 ^b^	16.38 ± 1.26 ^b^	12.02 ± 0.80 ^b^	3.79 ± 0.49 ^a^	2.75 ± 0.31	18.56 ± 1.14 ^b^	34.94 ± 2.03 ^b^
RO-control	5.73 ± 1.10	2.44 ± 1.64	8.17 ± 2.74	14.59 ± 0.39	24.25 ± 6.67 ^ab^	5.79 ± 1.15 ^b^	1.46 ± 0.35 ^b^	31.49 ± 7.47 ^b^	46.08 ± 7.08 ^b^
RO-RT	3.18 ± 1.77	3.29 ± 1.68	6.47 ± 2.22	19.17 ± 2.44	34.21 ± 3.38 ^a^	12.59 ± 0.85 ^a^	3.85 ± 1.13 ^a^	50.65 ± 5.07 ^a^	69.82 ± 7.48 ^a^
RO+4	2.88 ± 1.08	2.61 ± 1.88	5.49 ± 2.84	14.06 ± 2.47	22.84 ± 6.69 ^b^	8.42 ± 3.22 ^b^	1.80 ± 0.64 ^b^	33.06 ± 10.53 ^b^	47.12 ± 12.87 ^b^
RO-20	2.96 ± 0.58	1.78 ± 0.93	4.73 ± 1.49	14.42 ± 2.47	20.44 ± 0.72 ^b^	6.62 ± 0.26 ^b^	1.79 ± 0.20 ^b^	28.85 ± 1.00 ^b^	43.28 ± 2.71 ^b^

Results are expressed as mean values ± standard deviation of three technical repetitions. Mean values labeled with a different superscript letter, within the same column and same cultivar are statistically different (Tukey’s test, *p* ˂ 0.05). In case there were no statistically significant differences the letters were omitted. EE C16—ethyl palmitate, EE C18—ethyl stearate, FAEE—fatty acid ethyl esters. C246 = C42 + C44 + C46, C0246 = C40 + C42 + C44 + C46.

**Table 3:** Concentration of phenolic compounds in Istarska bjelica (IB) and Rosinjola (RO) monovarietal virgin olive oils obtained from fresh fruits immediately after harvest (control) and oils obtained from fruits stored seven days at three different storage temperature (RT—room temperature, +4 °C and −20 °C) prior to production.

Phenolic Compounds (mg/kg)	IB-Control	IB-RT	IB+4	IB-20	RO-Control	RO-RT	RO+4	RO-20
*Simple phenols*								
hydroxytyrosol	9.30 ± 0.96 ^b^	27.50 ± 6.48 ^a^	20.15 ± 0.90 ^a^	7.31 ± 2.83 ^b^	10.76 ± 1.51 ^b^	31.47 ± 2.62 ^a^	22.25 ± 5.72 ^a^	20.93 ± 5.40 ^ab^
tyrosol	7.67 ± 0.73 ^c^	40.16 ± 8.53 ^a^	20.22 ± 1.62 ^b^	11.42 ± 2.36 ^bc^	2.13 ± 0.26 ^b^	9.12 ± 3.53 ^a^	8.16 ± 2.07 ^ab^	3.62 ± 3.09 ^ab^
hydroxytyrosol acetate	1.14 ± 0.39 ^b^	2.86 ± 0.60 ^a^	2.01 ± 0.18 ^ab^	1.11 ± 0.27 ^b^	0.87 ± 0.24	1.31 ± 0.22	1.20 ± 0.09	0.85 ± 0.14
vanillin	0.20 ± 0.02	0.19 ± 0.02	0.21 ± 0.01	0.20 ± 0.03	0.31 ± 0.04	0.25 ± 0.07	0.17 ± 0.06	0.20 ± 0.09
Total simple phenols	18.30 ± 0.88 ^c^	70.72 ± 15.19 ^a^	42.58 ± 0.53 ^b^	20.05 ± 5.36 ^c^	14.08 ± 2.03 ^b^	42.15 ± 5.87 ^a^	31.78 ± 7.82 ^a^	25.60 ± 8.27 ^ab^
*Secoiridoids*								
secologanoside	0.34 ± 0.09	0.21 ± 0.21	0.15 ± 0.18	0.24 ± 0.13	0.13 ± 0.14	0.18 ± 0.19	0.17 ± 0.08	0.26 ± 0.24
elenolic acid glucoside (isomer)	1.99 ± 0.46 ^a^	0.74 ± 1.13 ^ab^	0.44 ± 0.62 ^ab^	0.16 ± 0.10 ^b^	0.43 ± 0.67	0.73 ± 1.17	1.28 ± 1.07	0.47 ± 0.70
3,4-DHPEA-EDA	120.02 ± 36.92 ^a^	29.58 ± 9.90 ^c^	84.78 ± 3.87 ^ab^	50.36 ± 4.75 ^bc^	73.30 ± 5.57 ^a^	48.63 ± 10.97 ^a^	58.65 ± 14.35 ^a^	35.30 ± 5.75 ^b^
oleuropein aglycone (isomer I)	62.34 ± 17.93	46.23 ± 3.49	52.90 ± 1.78	44.50 ± 9.08	152.05 ± 6.27 ^a^	98.64 ± 16.17 ^bc^	111.01 ± 7.86 ^bc^	73.26 ± 11.66 ^c^
*p*-HPEA-EDA	49.03 ± 8.82 ^a^	31.47 ± 5.70 ^b^	47.18 ± 2.80 ^a^	35.86 ± 1.82 ^ab^	11.24 ± 0.66 ^a^	10.13 ± 1.65 ^ab^	9.85 ± 2.20 ^ab^	6.79 ± 0.54 ^b^
oleuropein + ligstroside aglycones I and II	51.52 ± 4.84 ^a^	28.75 ± 5.73 ^b^	35.64 ± 2.18 ^b^	33.31 ± 3.16 ^b^	14.55 ± 0.60 ^a^	10.27 ± 0.99 ^bc^	12.05 ± 1.81 ^ab^	8.02 ± 1.61 ^c^
oleuropein aglycone (isomer II)	91.19 ± 15.37 ^a^	60.43 ± 2.27 ^b^	83.55 ± 4.17 ^ab^	69.99 ± 8.19 ^ab^	68.50 ± 2.39	72.68 ± 8.09	59.62 ± 5.95	62.16 ± 0.99
ligstroside aglycon (isomer III)	1.61 ± 0.21 ^b^	2.72 ± 0.78 ^ab^	3.59 ± 0.98 ^a^	4.11 ± 0.05 ^a^	1.41 ± 0.14 ^a^	1.52 ± 0.04 ^a^	1.53 ± 0.37 ^a^	0.80 ± 0.09 ^b^
oleuropein aglycone (isomer III)	9.93 ± 2.77	11.37 ± 1.23	14.87 ± 2.44	15.87 ± 3.04	6.33 ± 0.36	7.82 ± 1.00	7.20 ± 1.59	5.32 ± 0.49
Total secoiridoids	387.98 ± 71.14 ^a^	211.49 ± 22.77 ^c^	323.09 ± 13.86 ^ab^	254.41 ± 21.81 ^bc^	327.94 ± 8.77 ^a^	250.60 ± 33.36 ^b^	261.35 ± 35.28 ^ab^	192.38 ± 19.30 ^b^
*Lignans*								
pinoresinol	12.46 ± 2.11 ^b^	17.35 ± 0.37 ^a^	17.94 ± 0.06 ^a^	10.96 ± 0.40 ^b^	3.11 ± 0.11 ^a^	2.96 ± 0.04 ^a^	3.03 ± 0.02 ^a^	2.72 ± 0.08 ^b^
acetoxypinoresinol	24.85 ± 1.60 ^a^	24.84 ± 0.51 ^a^	25.80 ± 1.01 ^a^	20.99 ± 1.08 ^b^	30.25 ± 0.55 ^a^	30.66 ± 0.78 ^a^	30.59 ± 0.95 ^a^	25.53 ± 1.64 ^b^
Total lignans	37.31 ± 3.56 ^b^	42.20 ± 0.89 ^ab^	43.75 ± 1.08 ^a^	31.96 ± 0.72 ^c^	33.36 ± 0.66 ^a^	33.62 ± 0.75 ^a^	33.62 ± 0.93 ^a^	28.25 ± 1.60 ^b^
*Phenolic acids*								
vanillic acid	0.96 ± 0.09 ^b^	0.86 ± 0.08 ^b^	0.94 ± 0.01 ^b^	1.20 ± 0.04 ^a^	1.03 ± 0.03	1.05 ± 0.06	1.06 ± 0.10	1.03 ± 0.73
*p*-coumaric acid	1.74 ± 0.21 ^b^	5.16 ± 1.17 ^a^	2.12 ± 0.11 ^b^	1.28 ± 0.05 ^b^	0.46 ± 0.01 ^c^	4.74 ± 0.26 ^a^	1.26 ± 0.06 ^b^	0.43 ± 0.10 ^c^
Total phenolic acids	2.70 ± 0.27 ^b^	6.02 ± 1.11 ^a^	3.06 ± 0.10 ^b^	2.48 ± 0.05 ^b^	1.49 ± 0.04 ^b^	5.79 ± 0.32 ^a^	2.33 ± 0.16 ^b^	1.46 ± 0.71 ^b^
*Flavonoids*								
luteolin	1.96 ± 0.25 ^a^	1.14 ± 0.05 ^b^	1.43 ± 0.10 ^b^	0.70 ± 0.05 ^c^	0.96 ± 0.11 ^a^	0.77 ± 0.04 ^a^	0.90 ± 0.08 ^a^	0.49 ± 0.09 ^b^
apigenin	1.01 ± 0.09 ^a^	0.60 ± 0.01 ^bc^	0.72 ± 0.05 ^b^	0.48 ± 0.02 ^c^	0.35 ± 0.04 ^a^	0.29 ± 0.02 ^b^	0.34 ± 0.01 ^a^	0.24 ± 0.04 ^b^
Total flavonoids	2.97 ± 0.33 ^a^	1.74 ± 0.07 ^b^	2.15 ± 0.16 ^b^	1.18 ± 0.05 ^c^	1.32 ± 0.15 ^a^	1.06 ± 0.05 ^a^	1.24 ± 0.09 ^a^	0.73 ± 0.11 ^b^
TOTAL PHENOLS	449.26 ± 74.39 ^a^	332.17 ± 9.24 ^b^	414.63 ± 14.35 ^ab^	310.06 ± 24.78 ^b^	378.19 ± 9.16 ^a^	333.22 ± 28.63 ^a^	330.32 ± 28.64 ^a^	248.42 ± 18.35 ^b^

Results are expressed as mean values ± standard deviation of three technical repetitions. Mean values labeled with a different superscript letter, within the same row and same cultivar are statistically different (Tukey’s test, *p* ˂ 0.05). In case there were no statistically significant differences the letters were omitted.
